# Learning experiences of adaptive experts: a reflexive thematic analysis

**DOI:** 10.1007/s10459-022-10166-y

**Published:** 2023-01-10

**Authors:** Joanne Kua, Winnie Teo, Wee Shiong Lim

**Affiliations:** 1 Department of Geriatric Medicine, Institute of Geriatrics and Active Ageing, Tan Tock Seng Hospital, 11 Jalan Tan Tock Seng, Singapore, 308433 Singapore; 2 Group Education, National Healthcare Group, Singapore, Singapore

**Keywords:** Adaptive expertise, Geriatric medicine, Intra-personal, Inter-personal, Learning experiences, Organizational, Reflexive thematic analysis

## Abstract

Whilst adaptive experts have well-researched beliefs and attitudes towards learning, what is unclear are the interactions that occur within the learning environment that constitute their learning experiences. The practice of geriatric medicine emphasises the interdisciplinary care of complex frail older adults. Our study sets out to understand the learning experiences of adaptive experts in geriatric medicine by examining how interactions at the intra-personal, inter-personal and organisational levels contributed to the development of adaptive expertise. We conducted an exploratory qualitative study through semi-structured interviews of 16 geriatricians experts from a tertiary hospital in Singapore. Data were analyzed via reflexive thematic analysis. The core essence of the learning experiences was described as a journey of ‘knowing when we do not know’, which was characterised by three themes: (i) Anchoring ethos of person-centric care where the experts drew upon their values to develop a holistic view of the patient beyond the medical domain, (ii) Enabling stance of being curious where their curiosity and openness to learning was nurtured through the practice of reflection, and with the benefit of time as a commodity and for development of expertise, and (iii) Scaffolding organisational culture of inquiry where an environment that is supportive of learning is built on the culture of psychological safety and the culture of mentoring. Taken together, our study highlighted the importance of interactions at the intra-personal, inter-personal and organisational levels in the learning experiences of adaptive experts.

## Introduction

Adaptive expertise is an essential skill that encompasses not just the ability to create knowledge but to leverage on this prior knowledge to flexibly adapt to tasks and novel situations more effectively (Gube & Lajoie, 2020; Schwartz et al., 2005). Out of the six paradigms of education that have been proposed for health professions education (Baker et al., 2021), the dynamic orientation of adaptive expertise is ontologically and epistemologically aligned with the Constructivism- Expertise domain, where knowledge is constructed as the learners are exposed to the various experiences through learning. This construct of knowledge is layered upon prior knowledge with the eventual aim of expertise development.

Adaptive experts embrace complexity and are excited about novelty. In addition, they possess cognitive flexibility, have high intrinsic motivation towards learning and adopt reflective practice regularly. This enables them to have a deeper understanding of the problem at hand allowing them to innovate solutions to solve the issue. (Kua et al., 2021). Of cardinal interest is how adaptive experts developed these traits. A recent study evaluated the impact of the learning environment and its influence on the development of adaptive expertise during residency (Regan et al., 2022). The authors examined the contributions of personal, social, organisational and physical/ virtual components and how these supported the environment for an adaptive expert to thrive. What remains unknown are the interactions that transpired within these four components that facilitated the development of adaptive experts. Learning experiences refer to any interactions, whether in traditional academic settings or non-traditional outside of school settings, in which learning can occur. These interactions arise through relationships that exist between the personal and the social world, mediated by forces of nature like maturation. It is through the negotiations between the construed world of the individual and the physical and social world that learning occurs. This implies that we will need to understand the values of the individual and how that has affected the way the social world has been engaged with, for us to be able to grasp its significance on how adaptive experts develop (Billet, 2009). In situated learning theory, great emphasis is placed on relationships and interactions with others to build understanding (Lave & Wenger, 1991). This social focus of education allows learning to take place through participation in social activities as learners pick up skills and behaviours synonymous with the community they are members of. Likewise, workplace learning literature identifies participation in work activities as a rich source of learning and maintenance of competence, as well as enculturation and socialisation as workers engage with organisational norms, culture and values (Billet, 2009). It is therefore of utmost importance to better appreciate how and why such interactions have contributed to learning experiences.

Learning experiences vary across different specialties within medicine, and indeed, may also vary depending on the culture of a department or institution. The practice of geriatric medicine emphasises the interdisciplinary care of complex frail older adults. (Ellis & Sevdalis, 2019). Geriatricians are trained to manage complex illnesses intertwined with multiple comorbidities, frailty and other syndromes in frail older adults (Lester et al., 2019). In addition, it is inadequate to adopt a solely disease-centric approach without considering the behavioural and psychosocial sources of complexity (Safford, 2015). Adaptive expertise is thus required for geriatricians to manage complex cases in increasingly complex care environments with innovative approaches (Cutrer et al., 2019). Research on adaptive experts in medical specialties have examined ways knowledge can be constructed (Mylopoulos and Regehr, 2007; Varpio et al., 2009; Kawamura et al., 2014) and how prior knowledge is foundational for future practice (Cristancho et al., 2013; Mylopoulos & Scardamalia, 2008; Mylopoulos et al., 2012). In contrast, not much is known about how interactions and relationships have influenced the acquisition and subsequent interpretation of such knowledge to develop into an adaptive expert.

Our study sets out to understand the learning experiences of geriatricians practicing in a tertiary hospital who regularly manage complex acutely unwell frail older persons in their clinical practice. We examined how interactions at the intra-personal, inter-personal and organisational levels helped shape the development of adaptive expertise.

## Methods

### Overview

This is an exploratory qualitative study based on the interpretivist ontology which acknowledges diverse realities of participants (Crotty, 2003). Epistemologically, the study employs a social constructivist stance, where meaning is co-constructed between researchers and our participants (Ng et al., 2019a, 2019b). The study explores the learning experiences of participants in their journey to becoming adaptive experts in the field of geriatric medicine. Data was collected from 16 semi-structured interviews with the participants and analysed via reflexive thematic analysis (Braun et al., 2019; Terry et al, 2017).

### Data collection

A purposive sampling strategy was employed to ensure participants were recognised for their clinical expertise in geriatric medicine. The majority (n = 14) were senior consultant geriatricians in two hospitals in Singapore, who were appointed as faculty members in the National Healthcare Group Residency Programme. Two participants were associate consultants who had been practicing geriatric medicine for less than five years. We intentionally sampled junior geriatricians to capture the perspectives of participants who were still on the journey towards developing adaptive expertise, in contrast to their senior colleagues who were already acknowledged as experts in the field.

The study team (JK, WSL and WT) developed an interview schedule focused on how experts recognized clinically complex and novel cases, strategies for management, and how the experiences that these experts had allowed them to develop this expertise. It was not explicitly informed to the participants that the research was studying their journey into becoming an adaptive expert. Instead, the interview started by asking participants to recall one or two cases that involved a high level of complexity or novelty, and then asking them to delineate the factors that made these cases complex. Participants were then asked about their strategies for managing such cases. Thereafter, they were asked to share how they developed the ability to i) identify the factors that contributed to complexity of a case; ii) manage a complex or novel case. Using a semi-structured interview approach enabled the study team to develop the interview schedule based on iterative analysis of the data; in addition, the interviewers used probes to encourage additional specificity and detail related to the participants’ experiences in order to guard against recall biases in the recounting.

The study was approved by the institutional review board at National Healthcare Group (DSRB no 2018/00166). All interviews were conducted with individual participants (by either JK or WT), in-person and lasted up to 1 h in duration. All interviews were consented prior to audio-recording and were transcribed verbatim. Interview data was supplemented by field notes taken by the interviewers that noted their impressions and possible assumptions about the participants, and their experiences of conducting the interviews.

### Data analysis

Because we were interested in the meanings that participants attributed to the experiences that helped them develop adaptive expertise in geriatric medicine, data was inductively analysed using reflexive thematic analysis, guided by the 6 steps outlined by Terry et al. (2017), and with consideration of our research questions. This approach was chosen as it enabled the identification of patterns across our entire data set, while also allowing for a theoretically-informed interpretation of the data.

The first step involved familiarisation of the data, where both JK and WT immersed themselves in the data, re-reading and making notes on the content of the interviews. Both JK and WT then coded inductively, using Microsoft Word or Excel to highlight relevant passages of text with a descriptive code. The authors coded independently, generating a diversity of codes, and thereafter met regularly to review and discuss codes. In addition to generating codes at the semantic (explicit) levels, for example: individual practices that shaped learning experiences, they also coded data at the latent (hidden) level, exploring implicit ways in which the learning experiences were constructed (for example “time” being referenced in two distinct ways). Following coding, the entire team began the collaborative process of developing, testing and reviewing candidate themes. This was an iterative process that involved returning to the interviews to test if the emerging themes worked in connection with the coded extracts as well as across the entire data set. The final step (producing the report) involved revisiting the research questions, coding extracts and definitions of themes generated, as well as making connections between the data and literature.

### Rigour

To ensure the quality of our study, the study team adhered to the six steps outlined in Terry et al. (2017). Data accuracy was ensured by checking transcripts with original audio files and member checking. Both JK and WT engaged in researcher triangulation: while each coded the data independently, they also met together to discuss their impressions of the data.

Regular meetings among the entire study team members were important for reflexive engagement to challenge interpretations that could arise from potential biases, to examine data from multiple perspectives and to iteratively guide development of the interview questions. To maintain credibility of the data, an interview schedule was used for all interviews, and updated regularly as questions were revised.

The study team comprised 2 geriatricians (JK and WSL) and a researcher-administrator (WT), all with experience and roles in medical education research. The experience of the geriatricians afforded the study team access to the participants, but their positions and assumptions were constantly and critically considered to ensure that their perspectives did not bias interpretations of the data.

## Results

A total of 16 geriatricians from two major hospitals in Singapore participated in our study (Table 1). While majority (n = 9) of them had more than 10 years of experience as geriatricians, two of the participants had considerably less experience (less than 5 years). The study team included the insights of these two relatively junior geriatricians in order to capture the complementary perspectives of clinicians who were actively acquiring adaptive expertise in their learning journey.

**Table 1:** Learning experiences of adaptive experts: journey of ‘knowing when we don’t know’

Theme	Description	Subthemes
Anchoring ethos of person-centric care	Seeing the patient holistically sets the foundation for the expert’s learning experience	The value of values
Enabling stance of being curious	Having an attitude of curiosity helps nurture an openness to learning	The practice of reflection The benefit of time
Scaffolding organisational culture of inquiry	Building a culture of psychological safety and mentoring supports continued learning	The culture of psychological safety The culture of mentoring

Thematic analysis revealed that learning experiences were conceptualised as salient events and influences in the journey towards becoming an adaptive expert. Underpinning the core essence of ‘knowing that you don’t know’ are the 3 main themes of: (i) Anchoring ethos of person-centric care, (ii) Enabling stance of being curious, and (iii) Scaffolding organisational culture of inquiry (Table 2).

**Table 2:** Demographic information of participants (n = 16)

Participant no:	Sex	Years practicing as Geriatrician	Other roles
1	F	More than 10 years	Administrative lead
2	M	More than 10 years	Educator, Administrative lead
3	F	More than 10 years	Educator, Administrative lead
4	F	More than 10 years	Administrative lead
5	F	More than 10 years	Administrative lead
6	M	More than 10 years	Administrative lead
7	F	6–10 years	Educator
8	F	Less than 5 years	Clinician
9	F	Less than 5 years	Clinician
10	M	More than 10 years	Educator, Administrative lead
11	F	More than 10 years	Educator, Administrative lead
12	M	6–10 years	Educator, Administrative lead
13	M	More than 10 years	Educator, Administrative lead
14	F	6–10 years	Administrative lead
15	M	More than 10 years	Research, Administrative lead
16	M	More than 10 years	Administrative lead

### Defining the adaptive expert in geriatric medicine: ‘knowing that you don’t know’

The defining feature of an adaptive expert in geriatric medicine was described by participants as the insight to recognise situations which transcend their circle of competence. This awareness that ‘they knew that they didn’t know’ was the prelude towards framing the issues to put together a working diagnosis and treatment plan for the case, which often involved seeking assistance from others with the necessary expertise. A critical first step involved the ability to distinguish between a ‘usual’ vis-a-vis a ‘novel’ or more complex presentation. This typically involves a case that does not follow the usual history, or with an ambiguous trajectory that is unpredictable and may not reveal itself at first glance.

I mean if you see a lot of what's conventional, right, you would straightaway recognise what is unconventional. What is atypical in the presentation that doesn't fit neatly into the usual categories? And that's when you need to recognise that you need help. (I3)

The experts recognised that when they were operating at the boundaries of their expertise with a complicated or novel case, they needed to gather and triangulate information from various perspectives before formulating a diagnosis and treatment plan, following which, they needed to constantly review their plans in accordance with new information. This constant integration of new knowledge into their current plans would then effectively extend their boundaries of expertise, thereby diminishing the areas where ‘they knew they did not know’.

When asked about the experiences that helped shaped and developed their ability to recognize, formulate a diagnosis and initiate a treatment plan for a complex case, the experts articulated a range of salient experiences, which are summarised in the table of themes (Table 2).

### Theme 1: Anchoring ethos of person-centric care

Previous studies allude to the impact on self as the adaptive expert interacted with the external environment in which one is situated (Regan et al., 2022). Similarly, as one participant shared, viewing the patient in a holistic manner beyond the biomedical domain functioned as an anchor of their learning journey: “see(ing) the patient not only from the medical perspective but also the functional and social perspective, and that moulds the entire clinical judgment” (I14). This ethos of person-centric care undergirds and pervades their learning experience of developing expertise.

### Subtheme: The value of values

It has been suggested that responsibility towards patients drives the learning process and hence sets the foundation for expertise development (Regan et al., 2022). The experts shared how values like empathy helped lay the groundwork for person-centric care. One participant explained how values like empathy enabled him to understand the needs of the patients, facilitated sense-making in the face of competing sources of information and guided prioritisation in the management of complex patients: ““(Empathy) helps us shape our approach to the patients, based on understanding ourselves and the needs of the patients …This helps us prioritise and organise (in) the chaos of information that we have for the patients.” (I13).

Beyond empathy, self-awareness and self-control are defining features which set the expert apart. The experts articulated the importance of recognising the impact of emotions (both positive and negative) such that one is able to strike a balance and not let emotions cloud one’s clinical judgment. One participant elaborated on how even empathy had to be sometimes kept in check: “I think being a balanced person is very important because doctors have feelings too… we can empathise and sympathise, but … when we empathise too much, our judgment will not be correct. If we empathise too little, we'll make the wrong decision.” (I4) Another participant articulated how temperance was important: “Sometimes if you're too 'gung-ho', then you might actually harm the patient. So it has always got to be tempered by temperance, by self-control.” (I11).

In the endeavour for person-centric care, besides empathy and self-control, good judgment was also cited by the experts. It refers to the ability to contemplate, integrate and act in a sound and discerning manner not just through the attainment of consummate medical knowledge or garnering the inputs of other colleagues, but also invoking common sense and practical understanding to yield the rich perspectives of deep insight.

We need to exercise judgment and not just fall back on the opinion of people. Yeah, I think we always need to exercise judgment and see if it fits with our own line of reasoning and decision-making. (I9)

### Theme 2: Enabling stance of being curious

The experts mentioned curiosity as a key attribute for continual learning. In fact, one participant commented that “this curiosity actually makes them learn much better” (I1). For them, curiosity motivated them to go beyond one’s work tasks, asking questions, reading up on cases and thereby being open to learning. As one participant shared, “if I see something very ‘curious’, I have to go and read…. curiosity is very important. If you don't know, better go and read.” (I12).

You have to have a good attitude, be prepared to go out of the ordinary, be curious to ask questions, always thinking of the patient, and trying to add on to medical knowledge and clinical knowledge. (I2).

This curiosity was also grounded in humility – acknowledging that “not everyone knows everything, so there is always a lack of knowledge” (I3). In the management of uncertain situations, humility was especially important as they sought help from peers and seniors, both within and outside the discipline: “But if you're stuck, it's also important to ask the specialist of the area or your colleague for help” (I15) The interactions with their peers and seniors generated curiosity which aids in the learning.

The experts elaborated that while some learners were naturally curious, curiosity should also be nurtured. One participant felt that as senior geriatricians, “we have to stir up the curiosity.” (I3) Another participant (I15) shared that “some people are just naturally open, curious, (and) determined to put things together. Others need to have it demonstrated and they need to practice.” The experts felt that curiosity as a stance enabling continuous learning could be nurtured through the practice of reflection and through allowing time for the maturation and development of learners.

### Subtheme: The practice of reflection

With curiosity as the bedrock, a key element of the learning experience of adaptive experts is the practice of reflection (Mahant et al., 2012; Mylopoulos et al., 2011). Our participants commented on how reflecting on their personal practice helped inform and improve management decisions for their patients. One participant explained how reflection facilitated measured decisions in clinical management by providing a platform to consolidate and integrate inputs from different perspectives: “if I were to micro-manage I might miss important things….yet if I (take a) birds’ eye view, I might miss details and therefore management may not be appropriate or adequate — so I have to calibrate myself.” (I9).

### Subtheme: The benefit of time

Many of the experts reflected on their journey of expertise development and shared the impact that time had in their development. They spoke of time in two ways: firstly, time as a precious commodity. As one participant elaborated, setting aside time within the daily grind was necessary to gather information, iteratively process and think through the uncertainties surrounding a complex case, “I find myself having to relook the whole picture at certain points in time….I may even have to go back a few times to look at the whole picture again “ (I5).

However, finding time within the busy clinical schedule was a challenge. One participant commented how the lack of time was a critical constraint when learning, “The ward round is like a business round. We need to finish all the patients by three hours, after that we have other things to do. So, if you really want to do this properly, you need to bring the senior residents through those complex cases, sit down and go through each case with them. And we don't have time.” (I10).

The second way in which the experts spoke about time was in relation to the process of developing clinical maturity: the passage of time was needed for clinicians to mature through gradual accumulation of their clinical experience. A participant reflected that clinical experiences, accumulated through many years of exposure to a wide variability of cases, enabled him to practice in a more holistic and patient-centred manner: “when we first started, we were trained with a lot of knowledge and a lot of science…But over time as our life journey progresses and we start to accumulate more experience, then I adjusted away from (being) science-focused to person-focused.” (I13).

### Theme 3: Scaffolding organisational culture of inquiry

Some of the participants were administrative leaders, in addition to being clinical experts (Table 1). Drawing on their administrative experience, they highlighted that the learning experience of an adaptive expert required not only individual-level attributes and habits (such as curiosity, asking questions) but also a supportive organisational culture which fostered inquiry and learning. In particular, they mentioned the critical roles played by psychological safety and mentoring.

### Subtheme: The culture of psychological safety

An organisational culture of inquiry plays a critical role in the learning experience of adaptive experts by facilitating meaningful interactions in an environment which maintains trust and psychological safety. Psychological safety refers to a person’s sense that the immediate environment is safe for interpersonal risk-taking (Edmonson, 1999). A participant highlighted that a sense of psychological safety is important to encourage discussions about complex cases: “Having an environment of psychological safety – that really helps a lot. Openness to discuss, feeling safe to discuss cases that you're not sure of – all these can build up confidence at the same time.” (I8) Another participant commented that while it was challenging to do so, placing emphasis on learning and not blame was critical to creating a safe environment for conversations whereby the expert could be vulnerable and not be judged: “You need an environment that is not punitive…a punitive culture will create a defensive culture. Understanding that discussion is for learning and not for criticising. …sometimes we find it very difficult because it seems that we are criticising the person, but we really need an environment that we feel safe to discuss a case.” (I11).

### Subtheme: The culture of mentoring

The experts mentioned that mentors play a crucial role in their learning experiences, both in terms of sharing clinical insights to help develop expertise and in role modelling expected norms and behaviour needed for expertise development. A participant elaborated that having senior clinicians share their perspectives allowed him to understand the reasons behind their clinical decisions: “(I learnt) from observing my seniors and having them share their thoughts about (case) management…. Because when someone shares, then you realise this is the way they're thinking, why they are doing certain things…. So it helps when seniors share their experience.” (I9).

Another participant shared how senior clinicians who had exemplified behaviours crucial to continual learning and development had set him on the path to becoming an expert himself: “I do what I do because my seniors did the same thing. It's not something that is new… Like Dr. XXX and Prof YYY… Yeah, these are very, very senior clinicians, and they tell me, 'Oh, you know, every day I read… I study one chapter of a particular textbook’, or something like that …It's a habit that they have developed, and they have passed it on to countless other juniors including myself. So, it's all role modelling. If your consultant can do this, and he's got a family and all that, there’s no excuse for us.” (I10).

In addition to having individual mentors, the experts also stressed that fostering a culture of mentoring was equally important. Having a pool of senior doctors who were able to provide support to ‘scaffold’ the learner would enable learning from various sources of expertise: “The department staff will have to “wrap” themselves around this person to cover all their blind spots… as they progress in their work.” (I8). Another participant elaborated that it was important to gather different perspectives from various senior clinicians before reaching a decision on a complex case: “(I ask) different consultants to get their perspectives and suggestions on how to move forward with this complex case. So there's always people that we can turn to and a consensus can be reached.” (I14).

## Discussion

This study aims to understand how learning experiences in the form of interactions at the intra-personal, inter-personal and organisational levels contributed to the development of adaptive expertise in the complex field of geriatric medicine. Previous research has clearly articulated the core philosophies and deliberate practices which adaptive experts display (Mahant et al., 2012). Our study builds upon the emerging body of evidence by examining the development of adaptive expertise through the learning experiences of geriatrician experts in a tertiary hospital. The core essence of the learning experiences was described as a journey of ‘knowing that you don’t know’, elaborated by the major themes of anchoring ethos of person-centric care, enabling stance of being curious, and scaffolding organisational culture of inquiry.

Being patient-centered is a valuable trait of an adaptive expert (Mahant et al., 2012; Mylopoulos et al., 2017). Our study establishes the key values elicited as the physician interacts with patients and their caregivers, and which drive person-centric care. Some of the core values mentioned are empathy, self-awareness and self-control. Empathy is described as having not just an ability to ‘feel’ with the suffering of the patient but also the cognitive ability to ‘put oneself in the shoes of the patient’ (Reiss, 2010). Physicians with greater empathy have improved medical outcomes (Mercer et al., 2016; Shanafelt et al., 2002) and experience greater professional satisfaction (Shanafelt et al., 2012). This ability to take the perspective of the patient has been frequently cited by our participants as the core reason they are able to set clear priorities in the management of patients no matter how complex the case is. Empathy forms the basis for emotion-based reasoning and the skillful utilisation of empathy connects the doctor to the patients and gives meaning to the interaction (Ekman & Halpern, 2015), resulting in better patient outcomes with improved engagement with medical care plans (Ekman & Krasner, 2017).

The application of temperance and self-control, is required to weigh the benefits and harms in clinical interventions and prioritise management based on moral and systems issues that arise (Pellegrino & Thomasma, 1993). The ‘wise’ physician manages not only the clinical observations and evaluations of the patient but also the psychosocial existential concerns of the patient and helps them navigate through the complex systems of care to fulfill their needs. Our participants articulated that temperance and empathy work synergistically. This complementary relationship is essential for adaptive experts as it prepares them for anticipating care needs and anchors them to what matters most to the patients. The adaptive experts resist the urge to rigidly apply their evidence-based knowledge but choose to shift the focus to what could make a difference to the care of the patient instead through the application of ‘evidence-balanced medicine’ (Lim & Ding, 2015). This is known as cognitive self-regulatory process and has been described to support how health professions translate knowledge into action (Kahlke et al., 2020; Kuhl & Beckmann, 1985).

Curiosity has been described as a key attribute of adaptive experts (De Arment et al., 2013). We propose that curiosity is further nurtured through interactions when discussing cases with peers and seeking help from seniors. Curiosity is integral to clinical reasoning (Redelmeier, 2005) as it helps develop good habits of reflective practice, critical thinking and a quest for new knowledge (Shulman et al., 1968). These metacognitive skills have been well-described in previous research on adaptive experts (Carbonell et al., 2014; Hayden et al., 2013; Mahant et al., 2012; Mylopoulos et al., 2011; Yoon et al., 2015). Our research suggests that it is epistemic curiosity that drives the interactions with the peers and seniors to increase their knowledge. Through these interactions, epistemic curiosity further develops to diversive curiosity which is the basis for the interest and openness to novelty. Epistemic curiosity has been associated with creative problem solving and creative performance which are all hallmarks of an adaptive expert (Hardy et al., 2017).

Reflection is defined as ‘active, persistent and careful consideration of any belief or supposed form of knowledge in the light of the grounds that support it and further conclusions to which it tends’ (Dewey., 1910). Reflection aims to transform the learner to produce deeper knowledge that influences one’s practice of medicine (Ng et al, 2019a, 2019b). It is a way of thinking and reasoning that pushes the learner beyond knowing about the ‘what’ to knowing ‘why’ (Mylopoulos et al., 2018).

In addition, time is required for expertise to develop (Kawamura et al., 2014). Educators need to be mindful to provide learners time to slow down. This helps to nurture their curiosity by encouraging curious reflection, as the learners critically examine their own assumptions as they view the case from another perspective (Dyche & Epstein, 2011). This critical examination of themselves and their competence can sometimes be disorienting to the learner, but a disorienting dilemma catalyses transformative learning (Laros, 2017). When guided with appropriate feedback techniques, for example using advocacy and inquiry (Loo et al., 2018), transformative learning results in perspective transformation as the learner critically reflects on his own assumptions and beliefs as they approach uncertainty within a complex healthcare system (van Schalkwyk et al., 2019).

Beyond the individuals and their immediate environment, our study recognizes the impact of the organisation on the development of an adaptive expert. An organisation’s culture is constituted by values and expectations that guide and inform the members of the organisations about accepted behaviours and practices. Adaptive experts value an organisation where there is psychological safety and an emphasis on a culture of learning instead of a culture of blame, such that concerns and viewpoints can be expressed without fear of embarrassment or ridicule or shame (Edmonson, 2018). The perception of psychological safety provides the scaffold for clinician experts to feel secure enough for interpersonal risk-taking when confronted with clinical ambiguity, be it seeking help when required, questioning the status quo, or having the assurance that mistakes will be worked on together as a source of learning instead of being treated as a crime to be punished or covered up (Torralba et al., 2020). A vibrant culture of mentoring is also helpful. Adaptive experts can role model to learners the humility to seek help when they encounter difficulties (Mahant et al., 2012; Mylopoulos & Reghr, 2007). They also play a critical mentoring role in nurturing curiosity within learners by stimulating them to develop deeper understanding of the issues through reflection and critical thinking. Interestingly, in the sensemaking process when deciding who to seek help from, learners tended to approach those whom they perceive as accessible and trustworthy (Hofmann et al., 2009). Taken together, in a learning organisation which places high premium on psychological safety and which embraces a culture of mentoring, learners can progress more readily in their journey of becoming an adaptive expert through the guided discovery of accessing their interconnected knowledge networks and having the courage to push boundaries through creativity and innovation (Bransford et al., 2000).

Our participants had characterised their learning experiences as salient events and influences in their journey towards developing adaptive expertise. This framing deviates from the way in which adaptive expertise is commonly presented in literature, as an activity that learners engage in (Mylopoulos & Reghr, 2007). However, our participants had also acknowledged that this journey was in itself dynamic, and they themselves were continually learning, being curious and reflecting on their practice. It was significant that many of the experts chose to talk about their experiences and activities in the present tense – indicating that they saw themselves as still on the journey to (continually) develop expertise, rather than travellers who had already reached ‘a state of nirvana’. “

Our study comprised only two geriatricians who practiced for less than 5 years. Even though they shared primarily similar views as the senior geriatricians, it is important to further understand the learning experience of junior adaptive experts in future research. Moreover, we were unable to account for the effect of maturation with time in our cross-sectional interviews. In addition, our study would benefit from the complementary perspectives of organisational leaders in positions of authority to ascertain whether their viewpoints with regards to interactions and enculturation at the organisational level are aligned with our adaptive experts. Future research should also study other inter-professional groups involved in the care of frail older persons to ascertain the transferability of our findings beyond the medical profession. Lastly, our study did not examine the influence of socio-cultural factors on interactions at the personal, social and organisational levels. In health professions education especially amongst doctors, senior clinicians are venerated and experts are traditionally regarded as authoritative figures and often maintain a veneer of ‘know-all’ respectability to avoid ‘losing face.’ In such situations, the core essence of ‘knowing that you don’t know” amongst adaptive experts in our study may be less applicable and may need to be tempered with rapport management in order to provide an environment with sufficient psychological safety for senior clinicians to role model the attributes of an adaptive expert (Hwang et al., 2003).

Taken together, our study highlighted the importance of interactions in the learning experiences of an adaptive expert. These interactions occur at the intra-personal, inter-personal and organisational levels to support the development of adaptive expertise. Interactions happening at the intra-personal and inter-personal levels emphasise the importance of virtues such as empathy, self-control and wisdom, and support the incorporation of humanities and related fields within the curriculum to enhance empathy amongst medical students (Fukuyasu et al., 2021). The catalytic effect of curiosity in the learning experiences affirms the critical role played by clinician experts in role-modeling the stance of curiosity and in optimizing interactions with learners to generate curiosity through the way they provide feedback using evidence-based techniques such as debriefing with good judgment. Our study also highlighted the significant contribution of the organisation to the development of adaptive expertise through the provision of an environment that is supportive of learning by building upon the culture of psychological safety and the culture of mentoring. Our study thus paves the way for future research in other socio-cultural settings and inter-professional groups to further explore the impact of learning experiences on the development of adaptive expertise.
